# *VigSatDB*: genome-wide microsatellite DNA marker database of three species of *Vigna* for germplasm characterization and improvement

**DOI:** 10.1093/database/baz055

**Published:** 2019-05-31

**Authors:** Rahul Singh Jasrotia, Pramod Kumar Yadav, Mir Asif Iquebal, S B Bhatt, Vasu Arora, U B Angadi, Rukam Singh Tomar, Sarika Jaiswal, Anil Rai, Dinesh Kumar

**Affiliations:** 1 Sam Higginbottom University of Agriculture, Technology and Sciences, Allahabad, Uttar Pradesh, India; 2 Centre for Agricultural Bioinformatics, Indian Council of Agricultural Research (ICAR)-Indian Agricultural Statistics Research Institute, New Delhi , India; 3 Department of Biochemistry and Biotechnology, Junagadh Agricultural University, Junagadh, Gujarat, India

## Abstract

Genus *Vigna* represented by more than 100 species is a source of nutritious edible seeds and sprouts that are rich sources of protein and dietary supplements. It is further valuable because of therapeutic attributes due to its antioxidant and anti-diabetic properties. A highly diverse and an extremely ecological niche of different species can be valuable genomic resources for productivity enhancement. It is one of the most underutilized crops for food security and animal feeds. In spite of huge species diversity, only three species of *Vigna* have been sequenced; thus, there is a need for molecular markers for the remaining species. Computational approach of microsatellite marker discovery along with evaluation of polymorphism utilizing available genomic data of different genotypes can be a quick and an economical approach for genomic resource development. Cross-species transferability by e-PCR over available genomes can further prioritize the potential SSR markers, which could be used for genetic diversity and population differentiation of the remaining species saving cost and time. We present *VigSatDB*—the world’s first comprehensive microsatellite database of genus *Vigna*, containing >875 K putative microsatellite markers with 772 354 simple and 103 865 compound markers mined from six genome assemblies of three *Vigna* species, namely, *Vigna radiata* (Mung bean), *Vigna angularis* (Adzuki bean) and *Vigna unguiculata* (Cowpea). It also contains 1976 validated published markers. Markers can be selected on the basis of chromosomes/location specificity, and primers can be generated using Primer3core tool integrated at backend. Efficacy of *VigSatDB* for microsatellite loci genotyping has been evaluated by 15 markers over a panel of 10 diverse genotype of *V. radiata*. Our web genomic resources can be used in diversity analysis, population and varietal differentiation, discovery of quantitative trait loci/genes, marker-assisted varietal improvement in endeavor of *Vigna* crop productivity and management.

## Introduction

Genus *Vigna* is one of the most important Fabaceae family crops, which is mostly found in tropical regions and warm temperate region of Asia and Africa (1), but some of its species are also found in America (2). Myanmar, India and Brazil are the top three dry bean-producing countries in the world with 5.2, 3.9 and 2.6 MT, respectively (3).

Genus *Vigna* has six subgenera, namely, *Ceratotropis*, *Vigna, Lasiosporon*, *Plectotropis*, *Haydonia* and *Sigmoidotropis* (4). Genus *Vigna* having more than 100 species is one of the most underutilized leguminous crop for human food and animal feeds, as only 10 species are domesticated (5). Mungbean (*Vigna radiata*), cowpea (*Vigna unguiculata)*, adzuki bean (*Vigna angularis*) and urdbean (*Vigna mungo*) are the major domesticated species. The vast genetic diversity in wild species of *Vigna* is its best attractive feature, and these wild species are highly tolerant to biotic and abiotic stress such as pests, diseases, drought, alkaline soil and high salinity. Since more than 90 species of this genus are well adaptive to very extreme ecological niches like marine beach, deserts and swamps, they could be a potential source of genomic resource for productivity enhancement (6).

Genus *Vigna* are known for its edible seeds and sprouts, which are highly nutritional, rich sources of proteins, relatively high in iron and high in vitamins, minerals and folate contents. This leguminous crop is known for its antioxidant properties and for having therapeutic attributes for many diseases like cancer, diabetes, Alzheimer’s diseases, arthritis and cardiovascular diseases (7). This crop also plays a role in improving soil fertility and texture and fixing atmospheric nitrogen (8). Intercrop rotation with *Vigna* is well known to improve yield production of the other crops (9).

Till now, genomes of three *Vigna* species *viz.* mungbean (*V. radiata*), adzuki bean (*V. angularis var. angularis*) and *V. unguiculata* (Cowpea) were sequenced*.* In genus *Vigna*, mostly are diploid (2n = 2x = 22), while in *Vigna reflexo-pilosa*, mostly are tetraploid (2n = 4x = 44). The genome size of all the *Vigna* species may vary from species to species ranging from 416 to 1394 Mb (9).

Simple sequence repeats (SSRs), also known as microsatellites, are the co-dominant markers and plays an important role in biological functions. It is present in the stretch of DNA sequences that contain 1–6 repeating units of nucleotides (10). These repeating units are present in both coding and non-coding regions of DNA, but it is more abundant in intron region (11). SSR markers play an extensive role in marker-assisted selection (MAS), association studies, species identification, fingerprinting, diversity evaluation, linkage mapping and gene mapping for crop improvement due to its co-dominant inheritance, multi-allelic nature, robust amplification and reproducibility nature. SSR became the most powerful and important technique for plant genetic studies (12, 13). SSR markers are highly polymorphic as it depends on the number of tandem repeats. There are high chances of dissimilarity in number of repeats between species or individuals, thus this leads to the chance of getting high polymorphism between two respective individuals (14).

Although reports are available with a limited number of SSR markers, no bulk discovery of such markers is reported by genome-wide SSR mining. More markers are required for MAS, genetic diversity, genetic linkage maps, association or comparative mapping and qualitative and quantitative traits of Genus *Vigna* (14–17).

Traditional methods of microsatellite discovery using genomic libraries are compromised with a number of markers besides time and cost. To overcome this problem, *in silico* approach can be used. *In silico* method has the advantage of predicting target-specific regions in genome, which can be more effective in developing molecular markers required for quantitative trait loci (QTL) and linkage mapping (18, 19). Present linkage map needs more molecular markers to increase the map density for fine mapping (20).

As per TRIPS (trade-related aspects of the intellectual property rights) agreement and intellectual property rights, plant breeders have their ownership of variety on the basis of distinctiveness, uniformity and stability (DUS) features, which can be used for assigning of new variety status as well as resolving legal varietal disputes (21). Microsatellite markers have been successfully used in supplementing DUS feature in variety identification for crops like wheat, barley, soybean, rice and potato (22). Such approach can also be used for varietal differentiation of different *Vigna* species. Since *Vigna* genus has more than 100 species and limited genomic resources are available from three species, extensive molecular mining of SSR markers and evaluation of their polymorphism with cross-species transferability can be a more pragmatic approach to cater the need of markers in hitherto untouched species.

Since flanking region of microsatellite markers are well conserved across closely related species, heterologous PCR primers of such locus can cater the immediate need of molecular markers most economically (23). Molecular markers developed from `focal species’ have been successfully used in `non-focal’ species for population differentiation and diversity analysis (24).

Molecular markers, especially SSRs, play an important role for mapping of genes, genetic analysis and QTL mapping. Many SSR markers have been developed for QTL mapping and linkage maps associated with important traits like resistance and yield to abiotic and biotic stress of various *Vigna* species like mungbean (14, 25, 8, 26) and cowpea (27).

Although a mega plant genome SSR database (PMDBase) contains 110 plant species that includes two *Vigna* species, namely, *V. radiata* (cv. VC1973A) and *V. angularis* (cv. Jingnong 6), it has several limitations like lack of automation for SSR mining, designing of primers, input query sequence limited to 30 Kb (11); thus, there is a need to develop user friendly SSR database. If all available genomic data of genus *Vigna* are used to develop such database, it can be a valuable genomic resource for several species of genus *Vigna*.

Present work aims at genome-wide mining of SSR markers in three species of genus *Vigna*, namely, *V. radiata* (mung bean), *V. angularis* (adzuki bean) and *V. unguiculata* (cowpea). It further aims at development of a web genomic resource having option for chromosome-wise SSR mining and primer designing for genotyping along with e-PCR-based polymorphism discovery. It also aims to provide annotated genic region SSR to be used as functional domain markers (FDMs).

## Materials and methods

### Data source

Genomic data of *V. radiata* (mung bean), *V. angularis* (adzuki bean) and *V. unguiculata* (cowpea) were retrieved from NCBI (Table 1). Apart from full genome assembly data, we also used other genomic data of *Vigna sp.* available in the public domain. *Vigna radiata* contains genomic data of two cultivars viz., VC1973A and RIL59 (bruchid-resistant recombinant inbred line) (9, 28), whereas we found three assemblies of *V. angularis* cultivars such as Jingnong 6, Kyungwonpat and IT213134 (29, 30, 31). *Vigna unguiculata* has only one assembly of IT97K-499-35 cultivar (32)*.* Out of six assemblies, three were sequenced chromosome-wise, i.e. VC1973A, Jingnong 6 and Kyungwonpat, and the rest are all scaffold- and contig-wise. Extensive literature survey was made to obtain validated SSR sequences in different species of genus *Vigna* to include in the web resource.

**Table 1:** Genomic data of different varieties of *Vigna* species used in the study

**Species**	**Cultivars**	**Accession no.**	**Assembly level**	**Size (Mb)**	**GC%**
*V. radiata*	VC1973A	GCA_000741045.2	Chromosome	463.638	34.40
*V. radiata*	RIL59	GCA_001584445.1	Scaffold	454.907	32.00
*V. angularis*	Jingnong 6	GCA_001190045.1	Chromosome	467.301	34.62
*V. angularis*	Kyungwonpat	GCA_001723775.1	Chromosome	444.439	31.77
*V. angularis*	IT213134	GCA_000465365.1	Scaffold	291.824	33.10
*V. unguiculata*	IT97K-499-35	GCA_001687525.1	Scaffold	695.046	13.6

### *In silico* SSR mining

SSRs were mined using perl script of MISA (MIcroSAtellite identification tool) with default parameters such as 10 repeating units for mono, 6 repeating units for di and 5 repeating units for tri, tetra, penta and hexa (33). Markers were obtained with their respective descriptive information such as marker type, repeat numbers, markers size, guanine-cytosine (GC) content, start and end position. Primer3core executable was integrated in this web genomic resource for the generation of primer pairs from flanking sequence of each locus. Primer generation parameters were as follows: melting temperature, 55–65°C; GC content, 40–70%; primer size, 18–27 bp; length and product size, 150–280 bp (34).

### Functional annotation of genic region SSR markers

Available two species of *Vigna*, namely *V. radiata* (cv. VC1973A) and *V. angularis* (cv. Jingnong 6) that are fully annotated for gene function were incorporated in genome browser in the database. This uses general feature format coordinates of each gene file over coordinates of mined SSRs and displays gene ID along with structural and functional details.

### *In silico* discovery of polymorphism and cross-species transferability

In-house perl script was used for prediction of *in silico* polymorphic SSR within the two species, namely *V. radiata* (VC1973A and RIL59) and *V. angularis* (Jingnong 6, Kyungwonpat and IT213134). Cross-species transferability was evaluated over genomic DNA sequence of *V. angularis* and *V. unguiculata* by e-PCR of microsatellite loci obtained from *V. radiata* cv. VC1973A. For this evaluation, loci were selected based on the following criteria: simple di-nucleotide repeat as they are expected to have more polymorphism due to higher probability of slippage in DNA replication (35) and loci having unique sequence of at least 100 bp in its flanking regions to avoid error in primer designing.

### Work flow of markers and polymorphism discovery

In order to mine the microsatellite repeat loci, provision has been made for pattern identification using perl script implemented in MISA tool (33). It mines SSR loci from specific genome assembly of selected species/variety by user. In order to get SSR primers for genotyping, Primer3core (34) executable is integrated at the backend. User can select target of mining as per their choice of chromosome-wise or contig-wise. In-house perl script was used to extract flanking length of 500 bp upstream and 500 downstream of the SSR loci in targeted sequence file.

The `Polymorphism’ tab is based on the allelic length polymorphism identification algorithms by counting the amplicon size (in basepairs). This search is based on two modules for primer selection, namely, `self-designed primers’ and `External primers’. The `self-designed primers’ search generates new primers. VigSatDb has the references genome of six assembles with user-friendly option for uploading of genotype file for polymorphic discovery. Both, forward and reverse primers along with marker are located in the genotype file. The markers with different product size in the reference as well as genotype file are considered to be polymorphic markers. The second module, `External primers’ is based on primers that are already published. An in-house perl script has been written to detect the polymorphic markers by searching existing primers of loci in the flanking region of reference genome file. Then the e-PCR product sizes are computed and displayed as polymorphic loci. Using R code, provision of simulated gel has been made. Moreover, all the results can be achieved as HTML files, downloaded directly or through mail.

### Database development

**Figure 1:**
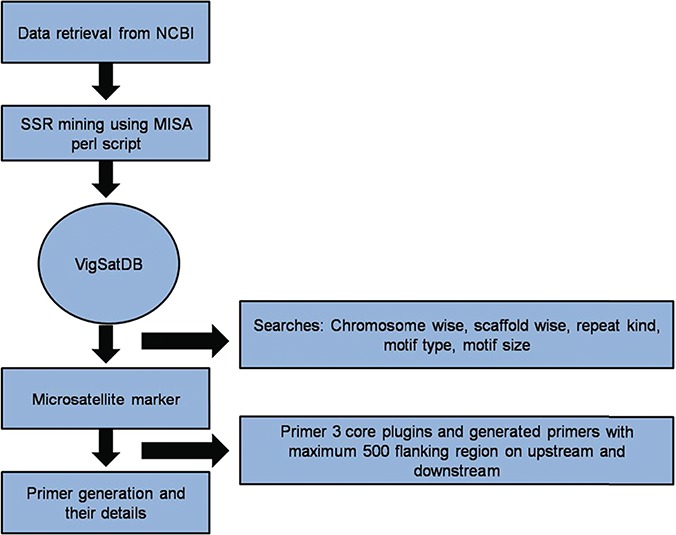
A schematic diagram of SSR marker discovery and primer generation.

*Vigna* microsatellite database (*VigSatDB*) a `*three tier architecture*’ relational database has been developed having client tier, middle tier and database tier. Data of predicted markers and experimentally validated published markers were stored in the backend in MYSQL database and launched on apache server. The web server contains seven tabs viz. Home, Microsatellite, Tools, Polymorphism, Statistics, Tutorial and Team. The database is further linked to web interface using open source PHP scripting language. Primer3core executable was incorporated into the database for primer generation. It gives the output having three sets of primers with their respective PCR product size, GC content, melting point and start position (Figure 1). Further, MISA scripts, Primer3core, NCBI local Blast (36) and The Generic Genome Browser (37) tools were integrated into the database for GUI-based web server. Moreover, in-house perl scripts for polymorphism search were also incorporated in the database for finding self design primers and external primers to evaluate published primers.

### Revalidation of microsatellite primers generated by *VigSatDB*

In order to check the efficacy of using *VigSatDb* for primer generation, *in vitro* validations were done. Fifteen primers were generated for microsatellite genotyping by PCR using a panel of ten genotypes of mungbean (*V. radiata*), namely NMK1512, SKM 1508, GSM1715, VIRAT, GAM5, GM-4, K-857, GJM1712, MEHA and VMS-15-1. These varieties were collected from Main Pulse Research Station, Junagadh Agricultural University, Junagadh, Gujarat, India. Young leaves were used to isolate DNA by CTAB method using protocol as described by Doyle and Doyle (38). The DNA concentration and purity of all genotypes were estimated spectrophotometrically using Picodrop (Picodrop Ltd Cambridge, UK).

### PCR-based genotyping of microsatellite loci

PCR reaction for all the microsatellite loci were carried out in a 20-μl PCR reaction mixture containing 4 μl 2.5 mM dNTPs, 1 μl of 20 ng DNA in 2.5 μl 10× Taq polymerase assay buffer, 1.5 μl MgCl_2_ and 1.5 U Taq polymerase enzyme (Sigma Aldrich, Germany) along with 1 μl (1 μM) each of SSR forward and reverse primers. SSR-PCR product was further analyzed on a capillary electrophoresis (QIAxcel Advanced System, QIAGEN, Germany) using QIAxcel DNA Screening Kit and the images were analyzed using QIAxcel ScreenGel Software.

## Results

### *In silico* SSR mining and analysis of its abundance

SSR loci were mined successfully using MISA. SSR loci details were also mined from the public domain literature. Web genomic resource was developed with provision to generate primers for genotyping of loci. A total of 876 219 putative microsatellite markers were mined having 772 354 simple and 103 865 compound repeats (Table 2). Genotype-wise motif types were obtained (Table 3).

**Table 2:** Distribution of SSR types in *Vigna* species

**Species**	**Total**	**Simple**	**Compound**
*V. radiata* cv. VC1973A	200 178	174 113	26 065
*V. radiata* cv.RIL59	200 642	173 405	27 237
*V. angularis* cv. Jingnong6	147 634	130 521	17 113
*V. angularis* cv. Kyungwonpat	143 109	126 601	16 508
*V. angularis* cv. IT213134	92 467	82 406	10 061
*V. unguiculata* cv. IT97K-499-35	92 189	85 308	6881
**Total Markers**	876 219	772 354	103 865

**Table 3:** Distribution of motif type found in each cultivar

**Unit size**	**VC1973A**	**RIL59**	**Jingnong6**	**Kyungwonpat**	**IT213134**	**IT97K-499-35**
**Mono**	127 648	127 845	86 204	78 016	54 256	59 935
**Di**	51 742	50 825	42 703	48 685	25 646	21 813
**Tri**	17 872	18 956	16 534	14 560	11 330	9260
**Tetra**	1827	1822	1212	1200	867	872
**Penta**	912	1003	734	414	231	187
**Hexa**	177	191	247	234	137	122

**Figure 2:**
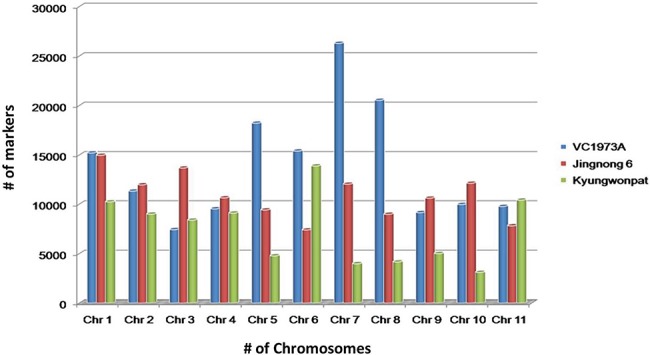
Chromosome-wise abundance of SSRs in *V. radiata* (cv. VC1973A), *V. angularis* (cv. Jingnong 6) and *V. angularis* (Kyungwonpat) varieties.

The genotype having chromosome-wise whole-genome data of all three species, *viz., V. radiata* (VC1973A), *V. angularis (*Jingnong 6) and *V. angularis (*Kyungwonpat), was used for the initial mining of SSR loci. Species and genotype-wise variation in abundance of SSR loci are shown in Figure 2.

In cv. VC1973A, maximum repeats were found on chromosome 7 (26 280), followed by chromosome 8 (20 521) and chromosome 5 (18 212). Chromosome 3 had minimum number of repeats (6579) having maximum SSR density per Megabase (Mb) (573.2). The least SSR density was found on chromosome 6 (411.3).

Although Jingnong 6 and Kyungwonpat cultivars were from the same species, at chromosome level substantial variation in SSR abundance was observed. In Jingnong 6, maximum markers were found on chromosome 1 (14 964), while minimum markers were observed on chromosome 6 (7391). In the case of cv. Kyungwonpat, chromosomes 6 and 10 were found to be with maximum (13 887) and minimum (3102) markers, respectively (Table 4). In the case of Jingnong 6, maximum and minimum SSR densities were found on chromosome 6 (381.8) and chromosome 8 (291.5), respectively. In the case of Kyungwonpat cultivar, maximum and minimum SSR densities were found on chromosome 5 (431.8) and 2 (317.9), respectively. In this study, mono-nucleotides and di-nucleotide repeats were found to be highly abundant, while penta-nucleotide and hexa-nucleotide repeats shared less proportion of SSR. AT and AG repeats were found to have maximum occurrence among di-nucleotide repeats in all the six genotypes, while AAT/ATT repeats were found to be most frequent among tri-nucleotide repeats.

**Table 4:** Chromosome-wise distribution of SSRs in *Vigna* cultivars VC1973A, Jingnong6 and Kyungwonpat

	*V. radiata*		*V. angularis*		*V. angularis*	
	VC1973A	Density per Megabase	Jingnong 6	Density per Megabase	Kyungwonpat	Density per Megabase
Chr 1	15 207	416.6	14 964	354.3	10 229	339.1
Chr 2	11 317	446.2	11 948	335.3	8989	317.9
Chr 3	7424	573.2	13 679	331.9	8383	336.8
Chr 4	9522	457.5	10 632	306.6	9097	332.3
Chr 5	18 212	489.8	9411	306.6	4771	431.8
Chr 6	15 399	411.3	7391	381.8	13 887	370.0
Chr 7	26 280	472.6	12 016	304.9	3976	422.0
Chr 8	20 521	448.7	8976	291.5	4156	321.7
Chr 9	9144	435.2	10 605	334	5007	377.6
Chr 10	9966	474.6	12 112	299.6	3102	401.8
Chr 11	9768	495	7808	293.6	10 407	421.5

### *In silico* discovery of polymorphism and cross-species transferability

Species-wise data on SSR loci polymorphism was generated successfully by e-PCR using genomic data of respective varieties. A total of 164 polymorphic loci were obtained between genotypes VC1973A and RIL59 of *V. radiata.* Similarly, in species *V. angularis*, the two sets of comparison between genotypes Jingnong 6 and Kyungwonpat and Jingnong 6 and IT213134 revealed 243 and 195 polymorphic loci, respectively (Supplementary Table 1).

Cross-species transferability of SSR loci was evaluated using *V. radiata* cv. VC1973A as focal species over two non-focal species, *V. angularis* and *V. unguiculata.* Maximum transferability across species was found in non-focal species *V. angularis* (2202) than *V. unguiculata* (2373). In order to obtain more SSR loci having cross-species transferability, the second species (*V. angularis*) was treated as focal species against non-focal species (*V. unguiculata*), which revealed 5882 loci (Supplementary Table 2). Since flanking region of SSR loci are evolutionary well conserved, computational prediction of such loci have practical significance. Such SSR loci can cater the need of molecular markers even in the absence of whole-genome sequence data. Present finding of cross-species transferable loci can be prioritized for use in diversity and population differentiation analysis of other species of genus *Vigna*, which are without whole-genome sequence data. Since more than 100 species of genus *Vigna* are available but genomic sequence for marker development is confined to just three species, present finding is of immense values.

### Revalidation of microsatellite primers generated by *VigSatDB*

**Figure 3:**
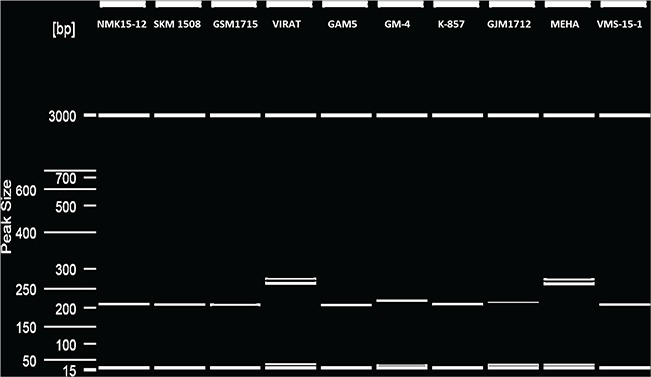
Microsatellite polymorphism detection in 10 genotypes of *V. radiata* in QIAxcel system.

PCR genotyping of 15 microsatellite loci in a panel 10 genotype was successfully obtained, demonstrating the efficacy of *VigSatDB*. Out of the 15 microsatellite loci, successful amplification was obtained for 14 loci. Figure 3 represents one of the representative gels showing amplification of microsatellite loci along with polymorphism. One locus could not be amplified, which may be due to PCR standardization method (Supplementary Table 3).

**Figure 4:**
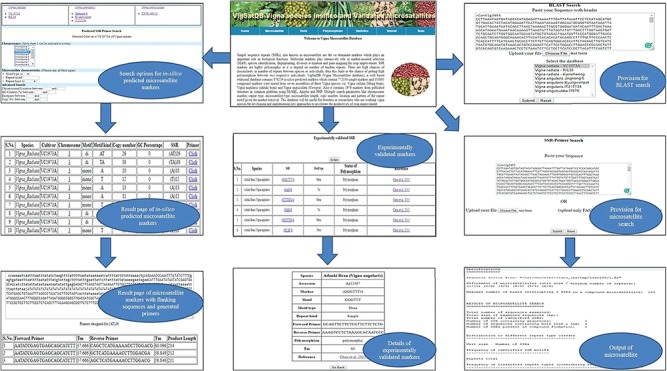
Interface of *VigSatDB* for various searches.

### Web genomic resources: *VigSatDB*

*VigSatDB* has been successfully developed using three-tier architecture*.* It contains a total of 876 219 SSR loci mined from whole-genome sequence data of three *Vigna* species represented by six different varieties. It also contains 1976 published SSR loci, which are already validated. This database has seven tabs viz. Home, Microsatellite, Tools, Polymorphism, Statistics, Tutorial and Team. The `Microsatellite’ tab provides two search options viz., *in silico* mined SSRs and experimentally validated SSRs. *In silico* mined SSR tab is used for mining of simple and compound SSRs of chromosome-wise or scaffold-wise genome assemblies along with motif type, motif kind, GC content, percentage, copy number and start position. Primer3core executable integrated at the backend can generate locus-specific primers of desired loci displaying their respective values of annealing temperature, product size and GC content. In order to use genic region SSRs as putative FDM, user has the choice to mine genic region SSRs selectively along with gene annotation. Using genome browser, user can get the details of gene name, accession number, descriptions, positions, strands, etc.

**Figure 5:**
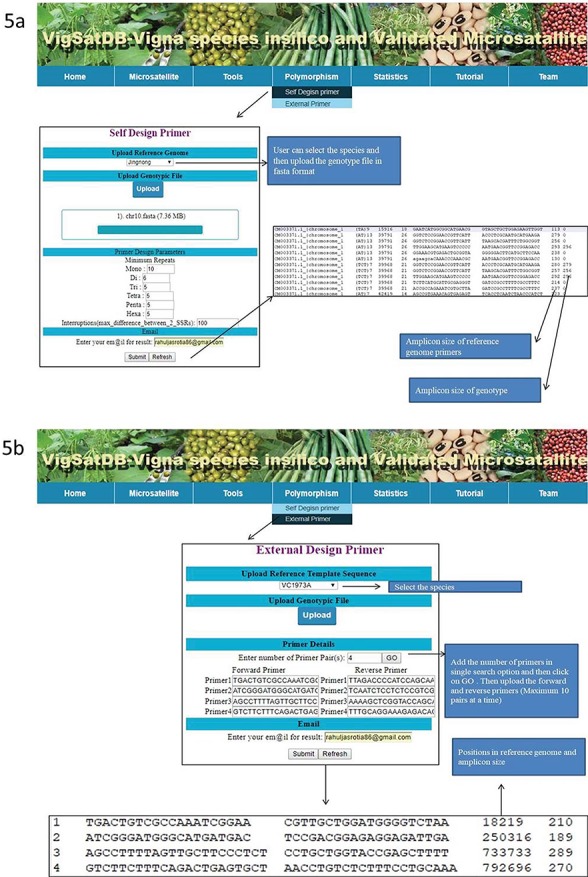
**(**A) *In silico* mining of SSRs and primer designing; (B)e-PCR evaluation of published primers using specific variety of *Vigna* species.

Experimentally validated SSR search tab exhibits the pre-existing validated markers from the literature. This can be searched by polymorphic, motif type, repeat motif and repeat kind. The results displayed contains details of its species, accessions, motifs, type, repeat kind, forward and reverse primers, annealing temperature along with reference of literature. Under the `Tool’ tab, provision has been made for SSR search and Blast search (Figure 4). The `Polymorphism’ tab provides two tabs viz., `Self Design Primer’ (for mining of SSRs along with primer generation) and `External Primers’ (for evaluation of published primers). The `Self Design Primer’ tab generates the new primers, and users can search polymorphic markers against six genomes. For this search, all six reference genomes of *Vigna* were already incorporated in the database, and users have to select the respective option and the input genotype file is in fasta format. For the best possible results, users can upload input genotype in chromosome-wise separately. The outputs of polymorphic markers are displayed on the screen or get the automated result mails. The option of external primer evaluation can be used advantageously to compare the published data with newly generated SSR data of varieties under investigation (Figure 5).

## Discussion

This first comprehensive microsatellite database of genus *Vigna* represented by three species co1tains 876 219 *in silico* predicted simple and compound markers and 1976 experimentally validated markers. This work clearly demonstrates that by *in silico* approach, mining of SSR can be done more efficiently (39). Since these markers are ubiquitously distributed over entire chromosomes, it can be a better representative in form of a molecular marker for genome variability analysis (40). Such approach has the advantage of location specificity over chromosome, thus it can be used as molecular marker of specific genes.

Flanking region SSR markers of targeted genes can be used in germplasm improvement program by MAS or introgression (41). Higher abundance of SSRs with respect to compound repeats was observed in all the genotypes. This is due to higher mutation rate of SSRs over compound repeat (42). All the six genotypes showed high abundance of mono-repeat, which might be due to inherent limitation of the Next Generation Sequencing (NGS) chemistry used in data generation (43). Similar higher abundance of di-nucleotide is also reported in other crops (44, 45). SSR relative density across all three species and their genotypes was found in 291–573 markers per Megabase, which is within the range of other reported crops like rice (370–490 markers per Megabase) and sugarbeet (341–671 markers per Megabase) (46).

A total of 200 178, 147 634 and 92 189 SSRs were mined from representative genotypes of three *Vigna* species such as *V. radiata* cv. VC1973A, *V. angularis* cv. Jingnong6 and *V. unguiculata* cv. IT97K-499-35, respectively. In species *V. radiata* cv. VC1973A, mono-nucleotides were the highest (127 648) and hexa-nucleotides were the lowest (177) in number. In *V. angularis* cv. Jingnong6, maximum numbers of SSRs were mono-type (86 204), while minimum number of SSR markers belonged to hexa-type (247). In *V. unguiculata*, mono-nucleotides were highest (59 935), while hexa-nucleotides were lowest (122) in number. In all the three species, mono-nucleotide repeats were most abundant followed by di- and tri-nucleotide repeats (Table 3).

Present finding of more than 875 000 putative SSR markers can be used for linkage mapping, identification of QTL/candidate genes and hybrid cultivar development. Similar use has already been reported in *Vigna* crop species for very limited few traits. For example, discovery of *cha* gene in *Vigna* is responsible for chasmogamous flower trait. This is highly relevant for molecular breeding to produce high yield in hybrid cultivar of *Vigna* (47). Similarly, *Vigna* SSR has also been used for PCR-based confirmation of successful production of interspecific recombinants of two species namely, mungbean (*V. radiata*) and mashbean (*V. mungo*) (48). SSR has been successfully used to identify tightly linked yellow mosaic virus resistance gene (49).

Variety characterization is based on phenotypic observation, which is very challenging to differentiate varieties with similar morphological characteristics. To overcome this traditional method, SSRs play an important role in variety identification, variety development, trait improvement, mapping, genetic studies and molecular breeding applications, product traceability, phylogenetic and taxonomic comparison (11, 50, 51). Very recently, 38 SSR markers of black gram (*V. mungo*) has been used to differentiate more than 30 varieties with high precision by rapid genotyping in DUS testing program of germplasm management (52). Similarly, 44 SSR markers have been used to differentiate five varieties of mungbean (*V. radiata*) (53). Using five set of SSR markers, 42 varieties (6 HYVs and 36 landraces) have been differentiated in mungbean (*V. radiata*) (22). Putative SSR markers present in *VigSatDB* can be a valuable genomic resource for similar varietal differentiation as ready to use primers for rapid genotyping are available along with *in vitro* validated SSR markers. Similar approach for varietal differentiation has already been reported in many other crops like sugarcane (54), sesame (55), capsicum (56), tea (57), barley (58) and eggplant (59).

Present set of markers can further be used for trait improvement against biotic and abiotic stresses. For example, in halophytic *Vigna marina*, SSR has been used to discover one major QTL controlling salt tolerance, and such QTL can be transferred in breeding program using flanking region SSR markers having allele size difference between donor and recipient varieties (60). These markers can be used for high-density linkage mapping and gene discovery required for specific trait improvement.

A large number of whole-genome assemblies of different plant species in the public domain are available, which provides the opportunity to study cross-species transferability in closely related species. This can be helpful in cloning candidate genes from different species and orthologous loci among different species (61). Also, *in silico* prediction of polymorphic markers can be useful in phylogenetic as well as diversity studies. *VigSatDB* putative markers can be used for germplasm improvement. Similar use in *Vigna* species has been reported with very limited markers (14). PMDBase is a comprehensive microsatellite database of 110 plant species, but has limitations that have been illustrated in Table 5. Our database can supplement the need of genomic resource for evolution and diversity analysis as reported in black gram (17).

**Table 5:** Comparison of PMDBase and VigSatDB databases

**Attributes**	**PMDBase**	**VigSatDB**
Number of *Vigna* species covered	**2**	**3**
Polymorphism prediction	**−**	**+**
Chromosome-wise search option	**−**	**+**
Motif-type search option	**−**	**+**
Repeat motif	**−**	**+**
Repeat kind	**−**	**+**
Genic and non-genic marker search by users’ choice	**−**	**+**
GC% content search option	**−**	**+**
Chromosomal location search option	**−**	**+**
Copy no. search option	**−**	**+**
Non-nuclear (mitochondrion and chloroplast)	**+**	**−**
Flexibility in primer designing	**−**	**+**
ePCR option for evaluation of published primers	**−**	**+**

Since genomes of most of *Vigna* sp. except *V. radiata*, *V. unguiculata* and *V. angularis* are yet to be sequenced, *VigSatDB* can be used as a genomic resource to cater the need of molecular markers required for QTL, linkage and genome mapping as well as germplasm improvement programs. Our finding of polymorphic SSRs and cross-species highly conserved SSRs can be used to characterize >90% species of genus *Vigna*. Such high transferability and their use in breeding, genetic, diversity and genomic studies are reported in crops like carrot species of Apiaceae family (62).

Although there are several known limitations in the utilization of cross-species SSR or heterologous SSR in diversity analysis (63), it is interesting to note in genus *Vigna* that the extent of SSR conservation is so high that even across genera (*Glycine max* and *Phaseolus vulgaris*) such conservation is reported in tune of more than one-fifth. Cross-species transferability of SSR loci in genus *Vigna* (*V. angularis*) is reported in tune of more than two-thirds in species like *V. umbellata, V. mungo, V. aconitifolia* and *V. radiata* (64). This extent of conservation has been in tune of more than three-fourths in *V. aconitifolia* and *V. reflexo-pilosa* (65). Higher rate of cross-species transferability of SSR loci in *Vigna* is due to their higher homology at genomic level; thus, they are much more useful in comparative genomics and genome mapping (8). Very safely it can be concluded that cross-species transferability of SSR loci is likely to be much more successful especially in crop like *Vigna*.

## Conclusion

We report here first comprehensive web genomic resource of genus *Vigna* covering three of its commercially important species. *VigSatDB* contains a total of 876 219 putative microsatellite DNA markers, which also includes 1976 validated published markers. A total of 10 457 polymorphic markers obtained by e-PCR across genotypes can be used for germplasm differentiation and diversity analysis saving *in vitro* cost and time required for *in vitro* polymorphic discovery. Our finding on cross species transferability of microsatellite locus among three different species of *Vigna* can be used to cater the need of molecular markers especially for more than 100 species of genus *Vigna* where there is no whole-genome sequence data even today. This genomic resource can be of immense use for the global community. It can be used for chromosome-wise microsatellite locus mining and primer designing for non-genic and genic FDM-SSR for rapid genotyping. It can also be used to accelerate polymorphism discovery by e-PCR most economically especially needed in future re-sequencing projects. It can be used not only for knowledge discovery research like QTL and gene mapping but also for marker-assisted breeding in *Vigna* germplasm improvement and management.

## Author’s contribution

P.K.Y., D.K., M.A.I., A.R., S.J. and R.S.J. conceived the theme of the study. R.S.J., M.A.I. and S.J. did the computational analysis of generated data. R.S.J., U.B.A. and V.A. contributed to database development. S.B.B. and R.S.T. did *in vitro* validation of markers. R.S.J., M.A.I. and S.J. drafted the manuscript. D.K., S.J., P.K.Y., M.A.I. and A.R. edited the manuscript. All authors read and approved the final manuscript.

## Supplementary Material

Supplementary_Table_1_baz055

Supplementary_Table_2_baz055

supplementary_Table_3_baz55

## Notes

**Database URL:** http://webtom.cabgrid.res.in/vigna_ssr/
